# Predicting the Risk of Human Immunodeficiency Virus Type 1 (HIV-1) Acquisition in Rural South Africa Using Geospatial Data^[Author-notes ciac069-FM1]^

**DOI:** 10.1093/cid/ciac069

**Published:** 2022-02-01

**Authors:** D Allen Roberts, Diego Cuadros, Alain Vandormael, Dickman Gareta, Ruanne V Barnabas, Kobus Herbst, Frank Tanser, Adam Akullian

**Affiliations:** Department of Epidemiology, University of Washington, Seattle, USA; Department of Geography, University of Cincinnati, Cincinnati, Ohio, USA; Heidelberg Institute of Global Health, Heidelberg University, Heidelberg, Germany; Africa Health Research Institute, KwaZulu-Natal, South Africa; Department of Epidemiology, University of Washington, Seattle, USA; Department of Global Health, University of Washington, Seattle, Washington, USA; Department of Medicine, University of Washington, Seattle, Washington, USA; Africa Health Research Institute, KwaZulu-Natal, South Africa; DSI-MRC South African Population Research Infrastructure Network (SAPRIN), Durban, South Africa; Africa Health Research Institute, KwaZulu-Natal, South Africa; Lincoln International Institute for Rural Health, University of Lincoln, Lincoln, United Kingdom; School of Nursing and Public Health, University of KwaZulu-Natal, Durban, South Africa; Centre for the AIDS Programme of Research in South Africa (CAPRISA), University of KwaZulu-Natal, South Africa; Department of Global Health, University of Washington, Seattle, Washington, USA; Institute for Disease Modeling, Bill & Melinda Gates Foundation, Seattle, Washington, USA

**Keywords:** HIV-1, risk prediction, South Africa

## Abstract

**Background:**

Accurate human immunodeficiency virus (HIV) risk assessment can guide optimal HIV prevention. We evaluated the performance of risk prediction models incorporating geospatial measures.

**Methods:**

We developed and validated HIV risk prediction models in a population-based cohort in South Africa. Individual-level covariates included demographic and sexual behavior measures, and geospatial covariates included community HIV prevalence and viral load estimates. We trained models on 2012–2015 data using LASSO Cox models and validated predictions in 2016–2019 data. We compared full models to simpler models restricted to only individual-level covariates or only age and geospatial covariates. We compared the spatial distribution of predicted risk to that of high incidence areas (≥ 3/100 person-years).

**Results:**

Our analysis included 19 556 individuals contributing 44 871 person-years and 1308 seroconversions. Incidence among the highest predicted risk quintile using the full model was 6.6/100 person-years (women) and 2.8/100 person-years (men). Models using only age group and geospatial covariates had similar performance (women: AUROC = 0.65, men: AUROC = 0.71) to the full models (women: AUROC = 0.68, men: AUROC = 0.72). Geospatial models more accurately identified high incidence regions than individual-level models; 20% of the study area with the highest predicted risk accounted for 60% of the high incidence areas when using geospatial models but only 13% using models with only individual-level covariates.

**Conclusions:**

Geospatial models with no individual measures other than age group predicted HIV risk nearly as well as models that included detailed behavioral data. Geospatial models may help guide HIV prevention efforts to individuals and geographic areas at highest risk.

## Teaser

HIV risk prediction models incorporating geospatial covariates identified individuals and geographic areas at high risk in South Africa. Simple models using age and local HIV prevalence had similar performance to models based on detailed demographic and seuxal behavioral measures.

Despite progress in expanding access to treatment and prevention services, human immunodeficiency virus (HIV) incidence in sub-Saharan Africa remains high. An estimated 870 000 people were newly infected in 2020, and annual incidence among adults exceeds 5 per 1000 population in several countries [1]. HIV prevention programs that prioritize services such as pre-exposure prophylaxis (PrEP) to individuals at the highest risk of infection will maximize benefits given available resources. The World Health Organization (WHO) has recommended that PrEP be offered to populations with HIV incidence of at least 3 per 100 person-years [2].

Accurate and efficient identification of individuals at high risk remains a key challenge for HIV prevention programs. Several model-based HIV risk prediction tools have been developed for women [3–5], men who have sex with men [6], serodiscordant couples [7], pregnant women [8], and the general population [9]. Existing tools rely primarily on age, sexual behavior, alcohol and drug use, and testing for sexually transmitted infections (STIs). These individual-level measures have several limitations. Behavioral risk factors are dynamic, require frequent reassessment, and are generally self-reported and thus prone to misclassification [10, 11]. Nucleic acid amplification testing for STIs is often not available outside of research studies or specialized clinics. The importance of specific individual risk factors may also vary across settings [12], which may diminish the performance of risk scores when applied in new populations [13].

Community-level measures of HIV prevalence, viral load, and treatment coverage offer an alternative approach to identifying individuals at elevated HIV risk. These measures are associated with HIV incidence [14–16] and act as proxies for local HIV transmission potential. Models based on geospatial measures may allow prioritization of prevention interventions to individuals and communities without the need to collect detailed behavioral data. However, existing HIV risk scores have generally not incorporated community-level HIV indicators, and the utility of geospatial measures in HIV risk prediction is unclear.

Using population-based data from a large prospective HIV cohort in South Africa, we developed and evaluated the predictive performance of gender-specific HIV risk prediction models. We compared the performance of models including both geospatial and individual-level covariates (full models) with models using only individual-level covariates (individual-level models) and models restricted to age and geospatial covariates (geospatial models). We also mapped the geospatial distribution of predicted risk from each model and compared the alignment with high incidence areas, as defined by the 2015 WHO PrEP guidelines (≥3 per 100 person-years ).

## METHODS

### Study Population

We used data from a large demographic surveillance system (DSS) run by the Africa Health Research Institute (AHRI) in the Hlabisa sub-district of KwaZulu-Natal, South Africa [17]. The southern surveillance area of the AHRI DSS, which has been followed since 2000, contains about 90 000 individuals in 11 000 households over a 438 square kilometer area. We excluded data from a northern surveillance area that was added in 2017. The study area is primarily rural with several peri-urban settlements and a single urban township. All households in the study area are contacted 3 times each year to interview the household head, who provides information on household attributes, births, deaths, and migration of residents. Since 2004, field workers have conducted annual surveys among household participants aged 15 years or older to assess demographics, sexual health, relationship history, and use of HIV prevention strategies. Participants then provide a dried blood spot (DBS) sample for anonymized HIV testing. Viral load testing has been conducted since 2011. In 2017, HIV prevalence among individuals aged 15–54 years was 20% for men and 41% for women, and overall HIV incidence was 2.3 per 100 person-years [18].

Eligible individuals for this analysis were aged 15–54 years with an initial HIV-negative test and at least 1 subsequent HIV test. For individuals with a subsequent positive HIV test, we randomly imputed a single seroconversion date from a uniform distribution defined between the last HIV-negative test and first HIV-positive test [19]. Follow-up time was right censored at the earliest of the last HIV negative test, first HIV positive test, date of death, or 55th birthday. To train the HIV risk prediction models, we used follow-up time occurring between 1 January 2012 and 31 December 2015 (development data set). We validated the models using follow-up time occurring between 1 January 2016 and 31 December 2019 (validation data set).

### Covariates

We constructed our models using a suite of time-varying individual-level and geospatial covariates. Individual-level predictors included 5-year age group, gender, marital status, education, employment, migration history, prior pregnancies or children, circumcision status, contraception use, number of sexual partners, and characteristics of the most recent partner. A full list of covariates and missingness frequency is available in the Supplementary Material (Supplementary Table 1). We estimated missing covariate values using multiple imputation by chained equations with 10 imputations [20]. Multiple imputation was carried out separately for the development and validation data sets to avoid bias from contamination between the two datasets [21]. Geospatial covariates included local estimates of HIV prevalence and population prevalence of detectable viremia (PPDV) [15], urban or rural designation, and distances from residence to the nearest roads, clinic, and schools. We produced annual estimates of local HIV prevalence and PPDV using moving 2-dimensional Gaussian kernels of a 3-kilometer search radius [22]. We chose the kernel radius a priori based on extensive previous work in the study area [14, 15, 23]. For each calendar year of follow-up time, we defined HIV prevalence and PPDV covariates by extracting the value of the prior year’s estimated surface at the coordinates of each individual’s residence.

### Model Development and Validation

We modeled time to seroconversion separately for men and women using Cox proportional hazards with least absolute shrinkage and selection operator (LASSO) penalties [24]. We selected optimal LASSO penalties via 10-fold cross-validated mean area under the receiver operating characteristic curve (cv-AUROC) evaluated at 1 year [25]. We fit 4 models with different covariate restrictions: all covariates (full model), only individual-level covariates, only age group and geospatial covariates, and only age group and local HIV prevalence. We finalized our models by fitting to the full development dataset using the optimal LASSO penalties and averaging the estimated hazard ratios across the 10 imputed data sets.

We validated each of the models by predicting hazard ratios in the validation dataset, and we evaluated model discrimination using AUROC. We evaluated model sensitivity by calculating the proportion of incident infections that occur within fixed percentiles of predicted risk for each model. We also calculated HIV incidence rates across quintiles of predicted HIV risk for each model.

### Geospatial Distribution of Risk

We evaluated how well our models identified geographic areas of high HIV risk by comparing the spatial distribution of predicted risk to the areas in which observed incidence exceeded 3 per 100 person-years. We estimated the spatial distribution of incidence by separately smoothing counts of cases and total person-time at risk using Gaussian kernels and then dividing the value from the cases surface by the value of the person-time surface across the study area. Because the number of incident cases is smaller and therefore leads to noisier geospatial estimates, we generated a single incidence surface across the study area by combining data from both men and women from 2012 to 2019 and increased the kernel bandwidth to 10 kilometers. We compared this surface to the spatial distribution of predicted risk over the same time period from each of the 4 models. For each modeled surface, we calculated the percentage of the area with incidence at least 3 per 100 person-years that was contained within the 20%, 40%, or 60% of the area with the highest predicted risk. We varied the incidence kernel bandwidth in sensitivity analyses. Geospatial analyses were conducted using ArcGIS (ESRI Inc, Redlands, USA), whereas all other analyses used R version 4.0.2.

### Ethics Approval

All participants provided written informed consent prior to the household-based interview and collection of dried blood spots. Approval for data collection and use was obtained from the biomedical and ethics committee (BREC) of the University of KwaZulu-Natal, Durban, South Africa (BREC approval number BE290/16).

### Role of the Funding Source

The funders of the study had no role in study design, data collection, data analysis, data interpretation, or writing of the report.

## RESULTS

The development datasets contained 9623 individuals (5910 women and 3713 men) with 841 seroconversions (679 among women and 162 among men) (Table 1). The validation data sets included 9933 individuals (6023 women and 3910 men) and 467 seroconversions (381 among women and 86 among men). Descriptive characteristics were similar between development and validation data sets, except for increases in educational attainment, circumcision, and contraception use (Table 2). Missingness was less than 5% for most variables but ranged as high as 46% for condom use at last sex (Supplementary Table 1). HIV incidence was 3.34/100 person-years in the development data set and 2.37/100 person-years in the validation data set, reflecting recent declines in incidence in the study area [18].

**Table 1.** Cohort Sizes and HIV Incidence Rates

		Development (2012–2015)	Validation (2016–2019)
Women	No. of individuals	5910	6023
	No. of seroconversions	679	381
	No. of PY	16 183	12 239
	Incidence rate (per 100 PY)	4.20	3.11
Men	No. of individuals	3713	3910
	No. of seroconversions	162	86
	No. of PY	9013	7436
	Incidence rate (per 100 PY)	1.80	1.16

Abbreviations: HIV, human immunodeficiency virus; PV, person-years.

**Table 2.** Descriptive Characteristics of Individuals in the Development and Validation Data Sets

		Men		Women	
		Dev.	Val.	Dev.	Val.
Age	15–19	36.1%	33.9%	23.8%	25.3%
	20–29	38.0%	37.5%	32.1%	32.0%
	30–39	11.7%	15.7%	14.0%	16.3%
	40–54	14.3%	12.8%	30.0%	26.5%
Education	Less than primary	7.1%	4.6%	8.1%	4.9%
	Primary	47.5%	39.6%	37.7%	31.0%
	Secondary or greater	45.5%	55.8%	54.2%	64.1%
Married		5.8%	4.3%	17.3%	15.3%
Employed		27.4%	22.7%	22.5%	17.0%
Prior outmigration		8.3%	13.5%	8.3%	11.7%
Ever had sex		59.3%	59.4%	76.5%	75.5%
Ever fathered children		24.7%	27.7%	-	-
Ever pregnant		-	-	64.4%	64.9%
Circumcised		7.4%	28.7%	-	-
Prior contraception use		-	-	19.6%	44.1%
≥1 partners in last 12 months		53.9%	52.7%	65.7%	65.2%
MRP casual^a^		30.1%	25.9%	18.4%	16.6%
MRP member of household^a^		20.6%	16.5%	39.9%	35.2%
Rural^b^		68.0%	66.0%	72.9%	70.0%
Mean local HIV prevalence^c^		24.6%	34.6%	23.8%	34.0%
Mean local PPDV^c^		15.3%	14.5%	14.7%	14.1%

Percentages are averaged across 10 imputed data sets.

Abbreviations: Dev, development data set (2012–2015); HIV, human immunodeficiency virus; MRP, most recent partner; PPDV, population prevalence of detectable viremia; Val, validation data set (2016–2019).

Evaluated among those reporting ever having sex.

< 400 residents per square km.

Estimated from a 2-dimensional Gaussian kernel with 3 km bandwidth.

The full models retained 38 predictors for men and 28 predictors for women (Supplementary Table 2). The strongest predictors included age group, marital status, circumcision (men), contraception use (women), sexual debut, number of partners in the last 12 months, number of current relationships (men), most recent partner residing outside of the household, and PPDV (men). The cv-AUROC for the full model in the development data was estimated as 0.74 (men) and 0.71 (women) (Supplementary Table 3). Models with covariate restrictions had cv-AUROC values ranging from 0.71 to 0.73 among men and 0.68 to 0.71 among women. In validation, the full models (AUROC = 0.72 for men and 0.68 for women) and models restricted to individual-level covariates (AUROC = 0.72 for men and 0.68 for women) had similar performance. Models restricted to age and geospatial covariates (AUROC = 0.71 for men and 0.65 for women) or age and HIV prevalence (AUROC = 0.68 for men and 0.64 for women) had slightly lower performance.

**Figure 1.**
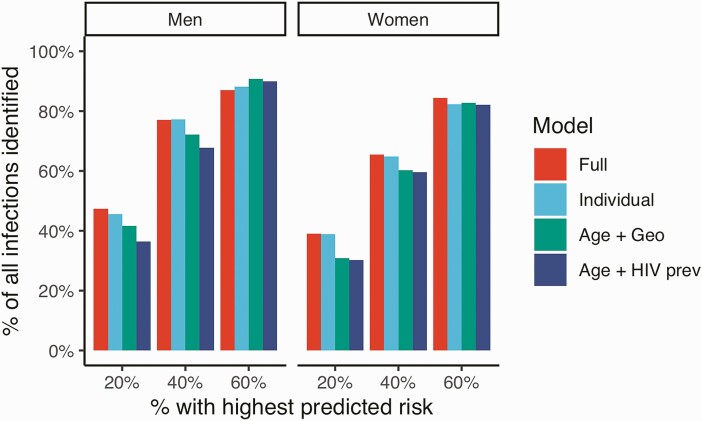
Percentage of new infections identified (sensitivity) within varying percentages of the population with the highest predicted risks. Estimated in the validation data set (2016–2019). Abbreviations: Age + Geo, only age group and geospatial covariates; Age + HIV prev, only age group and local HIV prevalence; Full, no covariate restriction; HIV, human immunodeficiency virus; Individual, only individual-level covariates;.

The sensitivity of the models at varying predicted risk thresholds is shown in Figure 1. Among the 40% of individuals with the highest predicted risk, the full model identified 77% of all new infections among men and 65% of new infections among women. Sensitivity at the 40% threshold was the same for models with only individual-level covariates (men: 77%, women: 65%) and slightly lower for geospatial models without individual-level covariates other than age (men: 68–72%, women: 60%). Incidence rates increased monotonically with increasing predicted risk quintiles (Table 3). Among men, the incidence rate in the validation data (per 100 person-years) among the 20% of individuals with the highest predicted risks ranged from 2.1 (95% confidence interval [CI]: 1.4–2.9) using the age + HIV prevalence model to 2.8 (95% CI: 1.9–3.6) using the full model. Among women, incidence in the highest predicted risk quintile ranged from 4.9 (95% CI: 4.0–5.7) using the age and geospatial covariates model to 6.6 (95% CI: 5.4–7.5) in the full model (Table 3). These incidence rates were between 5 and 11 times higher than the incidence rates in the lowest predicted risk quintile among women and 7–9 times higher among men.

**Table 3.** Incidence Rate (per 100 Person-Years) in Validation Data Set by Quintiles of Predicted Risk

		Full	Individual	Age + Geo	Age + HIV Prev
Men	Predicted risk quintile				
	1 (low)	0.3 (0, .5)	0.3 (0, .6)	0.3 (0, .6)	0.3 (0, .5)
	2	0.5 (.1, .9)	0.4 (.1, 0.8)	0.3 (0, .5)	0.3 (0, .5)
	3	0.6 (.1, 1.0)	0.6 (.2, 1.0)	1.0 (.5, 1.6)	1.3 (.7, 1.9)
	4	1.7 (1.0, 2.5)	1.8 (1.0, 2.5)	1.7 (1.0, 2.3)	1.8 (1.1, 2.5)
	5 (high)	2.8 (1.9, 3.6)	2.8 (1.8, 3.7)	2.4 (1.7, 3.2)	2.1 (1.4, 2.9)
Women					
	1 (low)	0.6 (.3, .9)	0.5 (.2, .8)	0.9 (.5, 1.2)	0.8 (.5, 1.2)
	2	1.9 (1.3, 2.5)	2.2 (1.6, 2.8)	2.0 (1.4, 2.5)	2.1 (1.5, 2.7)
	3	3.0 (2.3, 3.8)	2.9 (2.2, 3.6)	3.3 (2.6, 4.0)	3.3 (2.6, 4.1)
	4	3.9 (3.0, 4.7)	4.1 (3.2, 5.0)	4.8 (4.0, 5.7)	4.6 (3.7, 5.4)
	5 (high)	6.6 (5.4, 7.5)	6.2 (5.1, 7.2)	4.9 (4.0, 5.7)	5.0 (4.1, 5.9)

Parentheses indicate 95% confidence intervals. Abbreviations: Age + Geo, only age group and geospatial covariates; Age + HIV prev, only age group and local HIV prevalence; Full, no covariate restriction; HIV, human immunodeficiency virus; Individual, only individual-level covariates.

**Figure 2.**
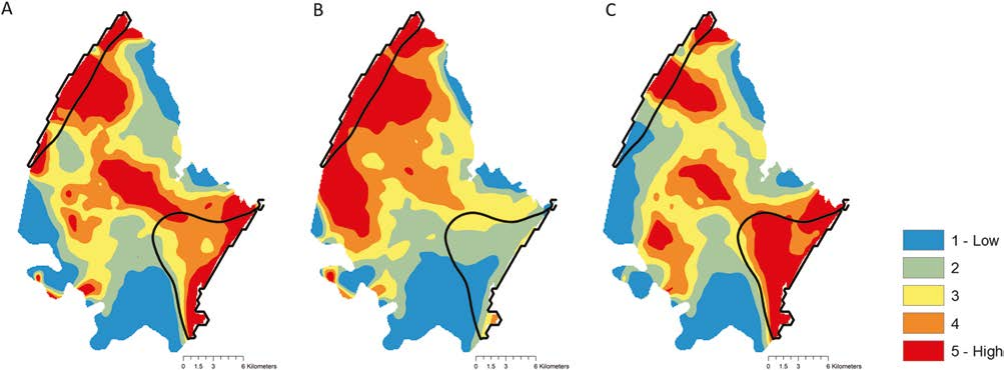
Geospatial distribution of predicted risk. *A*, Full model (no covariate restriction); *B*, only individual covariates; *C*, only age group and geospatial covariates. Maps include predictions for both men and women from 2012 to 2019. Model predictions were spatially smoothed using a 2-dimensional Gaussian kernel. Colors indicate quantiles of spatially smoothed model predictions. Solid lines enclose areas with estimated incidence ≥ 3 per 100 person-years.

The geographic distribution of predicted risk from models incorporating geospatial covariates aligned much more closely to the geographic distribution of observed HIV incidence than predictions from models using only individual-level covariates (Figure 2). The 20% of the map with the highest predicted risk captured 60% of the area with high incidence (≥3/100 person-years) when using the full model and 59–60% when using the models with only age and geospatial covariates (Supplementary Figure 1). In contrast, the geospatial distribution of predicted risk using the individual model did not align closely with incidence, and the 20% of the map with the highest predicted risk only accounted for 13% of the area with high incidence. These results were robust to varying the incidence kernel bandwidth (Supplementary Figure 2).

## DISCUSSION

We developed and validated human immunodeficiency virus type 1 (HIV-1) risk prediction models using a suite of individual-level and geospatial covariates to identify men and women at very high risk for HIV acquisition in rural KwaZulu-Natal, South Africa. Men and women within the top 20% of predicted risk had incidence rates of at least 2.1 and 4.9 per 100 person-years, respectively, across all models. Simple models using only age group and geospatial covariates (including a model with only age and local HIV prevalence) predicted individual-level risk of HIV infection nearly as accurately as models that included demographic, sexual behavioral, and socioeconomic predictors. Furthermore, spatially smoothed predictions from models that included geospatial covariates aligned closely with high incidence areas while predictions from models with only individual-level covariates did not.

Few HIV risk prediction models have incorporated geospatial measures, but those that have considered HIV prevalence have consistently found it to be predictive. An analysis from the communities in the vicinity of Rakai District in rural Uganda found that a 1 percentage point increase in community HIV prevalence was associated with a three percent increase in the hazard for HIV acquisition [26]. A risk score developed from the ECHO study, which used data from 9 South African sites spanning 5 provinces, found site-level HIV prevalence to be one of the strongest predictors of HIV risk [4]. Our analysis, which used micro-scale spatial variation in HIV prevalence and community viral load, extends these findings to a much smaller geographic scale, allowing identification of “corridors of HIV transmission” with high incidence [27]. Although community viral load has been strongly associated with HIV incidence in multiple settings [15, 16], georeferenced viral load data is not widely available, and we found that predictive performance was largely maintained in a model with only age and HIV prevalence. This result may reflect that PPDV is a function of HIV prevalence, and the 2 measures tend to be closely correlated in settings with low antiretroviral therapy (ART) coverage such as AHRI (51% among women and 38% among men in 2017) [18]. However, as ART scales up, PPDV may become a more sensitive measure of local transmission potential.

Risk scores based on detailed clinical and sexual behavior measures rely on accurate reporting of sensitive and potentially stigmatizing behaviors. Such approaches are best suited to clinical settings with sufficient resources for lengthy individual-based assessment. Our results indicate that similar predictive performance may be obtained from models using age group and smoothed HIV test results from population-based surveys. These models enable HIV prevention strategies focused more on geographic context than on individual risk behaviors. Additional investment in routine population-based HIV and viral load testing could facilitate resource prioritization to communities with the highest need.

Although our model identified individuals and geographic areas at the highest risk, incidence was still elevated among women even at lower predicted risk thresholds. Women in the second lowest quintile of predicted risk using the full model experienced an incidence rate of 1.9/100 person-years between 2016 and 2019, and women in the middle quintile had an incidence rate of 3/100 person-years. Our results indicate that, in a hyperendemic South African setting, incidence was high even among women who reported few individual-level risk factors. Similar gender disparities have been demonstrated across sub-Saharan Africa [28], and our findings underscore the continuing need for widespread coverage of combination HIV prevention services, especially for women.

Our analysis has several strengths. We used a robust internal validation strategy on a large dataset to develop our models, and we validated our predictions on data from subsequent years that were not used in model development. This validation strategy was chosen to estimate performance in prospective prediction, and our models performed well despite temporal changes in incidence and risk factor distribution. For future predictions, the models can easily be retrained using the same methods on an updated dataset. Additionally, our suite of models with different covariate restrictions allows for prediction in a variety of settings with different data availability. Models based solely on individual-level covariates could be applied in a clinical setting where geospatial covariates may not be available. In contrast, models based on age and geospatial covariates could guide geographic prioritization of interventions, such as focused community HIV testing campaigns, home-based ART delivery, or community PrEP services [29–31]. Approaches that combine both geographic and individual factors may be needed to achieve both widespread coverage to those at risk, as well as efficient resource allocation. Additional research is needed to optimize implementation of risk prediction tools in low-resource settings.

Our analysis also has limitations. Some of the sexual behavior variables had high amounts of missing data, and we lacked other indicators that have been previously incorporated into risk scoring tools, such as alcohol use, whether sex partners provide financial support, and whether sex partners have other partners [3]. Therefore, our models based on individual-level covariates may have lower performance than ones trained on richer data. We did not have STI testing, which has been incorporated into risk scores trained using clinical trial data but is not widely available, even in routine clinical care settings. We did not compare other algorithms for training our predictive models. Even so, the predictive performance of our individual-level covariate models (AUROC 0.68–0.71) was similar to the performance of models developed in other sub-Saharan Africa settings (AUROC 0.67–0.73) [3, 4, 9]. We also did not validate our models using data from other settings, so the generalizability of our findings is uncertain. Further research could evaluate predictions in other locations with routine population-based HIV surveillance at small spatial scales, such as ALPHA Network sites or universal test-and-treat trial locations [32, 33]. Additionally, small area HIV prevalence estimates have been generated for all of sub-Saharan Africa [34]; however, these estimates derive from sparser data, and their predictive performance at small geographic scales is unknown. Additional investment in population-based surveys and surveillance may be needed to improve estimates of local HIV prevalence and viremia in other settings. In addition to informing HIV risk prediction, these measures are directly applicable to HIV treatment targets and would have tangible benefits to ART programs [35].

In summary, we developed and validated a suite of risk prediction models informed by individual-level and geographic covariates that identified individuals and geographic areas at high risk for HIV acquisition. These results may guide efforts to prioritize HIV prevention resources to maximize impact.

## Supplementary Data

Supplementary materials are available at *Clinical Infectious Diseases* online. Consisting of data provided by the authors to benefit the reader, the posted materials are not copyedited and are the sole responsibility of the authors, so questions or comments should be addressed to the corresponding author.

ciac069_suppl_Supplementary_Material

## Notes

***Acknowledgments.*** The authors thank the AHRI staff for their involvement in data collection and data management, as well as all research participants for contributing their time, survey responses, and biological samples. They acknowledge Clinton Collins from the Institute for Disease Modeling (Seattle, Washington, USA) for providing computational support. The data sets used in this analysis are contained in the AHRI data repository (https://data.ahri.org/index.php/home). To access the licensed data sets, the applicant must agree to the terms and conditions of use by completing an application for access to a licensed data set. This request will be reviewed by the AHRI Data Release Committee, who may decide to approve the request, to deny access to the data, or to request additional information from the applicant.

***Financial support.*** The AHRI’s Demographic Surveillance System is funded by the Wellcome Trust (grant number 201433/Z/16/Z) and the South Africa Population Research Infrastructure Network (funded by the South African Department of Science and Technology and hosted by the South African Medical Research Council). This work is based on research funded in part by the Bill & Melinda Gates Foundation, including models and data analysis performed by the Institute for Disease Modeling at the Bill & Melinda Gates Foundation. D. A. R. received support from the Infectious Diseases Society of America Grants for Emerging Researchers Mentorship Program and the US National Institute of Mental Health (NIMH) (grant number F30MH122300). This research was also supported by the University of Washington/ Fred Hutch Center for AIDS Research, a National Institutes of Health (NIH)-funded program under award number AI027757, which is supported by the following NIH Institutes and Centers: National Institute of Allergy and Infectious Diseases (NIAID), National Cancer Institute (NCI), NIMH,  National Institute on Drug Abuse (NIDA), National Institute of Child Health and Human Development (NICHD), National Heart, Lung, and Blood Institute (NHLBI), National Institute on Aging (NIA), National Institute of General Medical Sciences (NIGMS), and National Institute of Diabetes and Digestive and Kidney Diseases (NIDDK).
