# Supplementary material for: Predicting the Risk of Human Immunodeficiency Virus Type 1 (HIV-1) Acquisition in Rural South Africa Using Geospatial Data
Source: Clin Infect Dis. 2022 Feb 1;75(7):1224–31. doi: 10.1093/cid/ciac069 (PMC9525068; doi:10.1093/cid/ciac069)

## Supplementary Material

**Table S1: Missingness frequency for variables considered in prediction models.** Only variables with at least 1% missingness shown.

| | Men | | Women | |
|---|---|---|---|---|
| | Development | Validation | Development | Validation |
| Lifetime number of partners | 31.3% | 32.0% | 32.5% | 24.7% |
| Used condom at last sex with MRP | 29.0% | 38.1% | 45.4% | 45.6% |
| MRP member of household | 17.9% | 28.0% | 19.5% | 25.0% |
| MRP casual | 17.7% | 27.8% | 19.3% | 24.7% |
| # partners in last 12 months | 17.6% | 26.4% | 18.6% | 23.3% |
| # current relationships | 17.1% | 25.5% | 17.6% | 21.6% |
| Prior contraception use | - | - | 5.3% | 14.1% |
| Ever had sex | 5.3% | 9.1% | 2.9% | 5.4% |
| Married | 3.4% | 4.2% | 2.5% | 3.5% |
| Ever pregnant | - | - | 2.3% | 4.7% |
| Has fathered children | 3.0% | 8.0% | - | - |
| Is circumcised | 2.8% | 10.9% | - | - |

**Table S2: Hazard ratios from coefficients retained in the final models.** Full = no covariate restriction; Ind = only individual-level covariates; Age + Geo = only age group and geospatial covariates; Age + HIV prev = only age group and local HIV prevalence; Ref = reference group; SES = socioeconomic status; PPDV = population prevalence of detectable viremia

| | | Men | | | | Women | | | |
|---|---|---|---|---|---|---|---|---|---|
| | | **Full** | **Ind** | **Age + Geo** | **Age + HIV prev** | **Full** | **Ind** | **Age + Geo** | **Age + HIV prev** |
| **Individual covariates** | | | | | | | | | |
| Age (Ref = 15-19) | 20-24 | 1.41 | 1.04 | 2.95 | 3.47 | 1.13 | 1.12 | 1.41 | 1.43 |
| | 25-29 | 1.79 | 1.12 | 5.26 | 6.32 | 1.08 | 1.06 | 1.38 | 1.40 |
| | 30-34 | 1.58 | 1.08 | 4.47 | 5.38 | 0.69 | 0.66 | 0.78 | 0.75 |
| | 35-39 | 0.99 | 1.00 | 2.51 | 3.00 | 0.56 | 0.52 | 0.52 | 0.45 |
| | 40-44 | 1.05 | 0.99 | 2.10 | 2.41 | 0.52 | 0.48 | 0.41 | 0.33 |
| | 45-49 | 0.83 | 0.96 | 1.47 | 1.62 | 0.54 | 0.51 | 0.41 | 0.33 |
| | 50-54 | 0.66 | 0.93 | 0.94 | 0.92 | 0.39 | 0.35 | 0.31 | 0.22 |
| Education (Ref = < Primary) | Primary | 1.49 | - | - | - | 1.27 | 1.28 | - | - |
| | Secondary + | 1.6 | - | - | - | 1.02 | 1.01 | - | - |
| Married (Ref = No) | | 0.40 | 0.65 | - | - | 0.50 | 0.49 | - | - |
| Employed (Ref = No) | | 0.87 | - | - | - | - | - | - | - |
| Asset quintile (Ref = lowest) | 2 | 0.99 | - | - | - | - | - | - | - |
| | 3 | 0.95 | - | - | - | - | - | - | - |
| | 4 | 0.80 | - | - | - | - | - | - | - |
| | 5 (highest) | 0.83 | - | - | - | - | - | - | - |
| SES quintile (Ref = lowest) | 2 | 1.20 | - | - | - | 1.00 | 1.00 | - | - |
| | 3 | 1.14 | - | - | - | 0.99 | 1.00 | - | - |
| | 4 | 1.23 | - | - | - | 0.99 | 0.99 | - | - |
| | 5 (highest) | 1.00 | - | - | - | 0.99 | 0.99 | - | - |
| Prior non-resident (Ref = No) | | 1.10 | - | - | - | 1.09 | 1.10 | - | - |
| Prior outmigration (Ref = No) | | 1.12 | - | - | - | 1.28 | 1.27 | - | - |
| Ever had sex (Ref = No) | | 4.5 | 4.61 | - | - | 1.34 | 1.33 | - | - |
| Ever pregnant (Ref = No) | | - | - | - | - | - | - | - | - |
| Has fathered children (Ref = No) | | 1.25 | - | - | - | - | - | - | - |
| Circumcised (Ref = No) | | 0.59 | - | - | - | - | - | - | - |
| Prior contraception use (Ref = No) | | - | - | - | - | 1.47 | 1.48 | - | - |
| # Partners in last 12 months (Ref = 0) | 1 | 1.03 | 1.01 | - | - | 1.15 | 1.17 | - | - |
| | 2+ | 1.42 | 1.11 | - | - | 1.98 | 2.13 | - | - |
| # Current relationships (Ref = 0) | 1 | 1.15 | 1.04 | - | - | 1.04 | 1.07 | - | - |
| | 2+ | 1.87 | 1.34 | - | - | 0.95 | 0.90 | - | - |
| MRP 5+ years younger (Ref = No) | | 0.97 | - | - | - | - | - | - | - |
| MRP 5+ years older (Ref = No) | | - | - | - | - | - | - | - | - |
| MRP casual (Ref = Regular) | | 0.77 | 0.99 | - | - | 1.08 | 1.14 | - | - |
| MRP member of household (Ref = No) | | 0.79 | 0.97 | - | - | 0.58 | 0.58 | - | - |
| MRP used condom last time (Ref = No) | | 0.92 | - | - | - | 0.99 | 0.98 | - | - |
| **Geospatial covariates** | | | | | | | | | |
| HIV prevalence (per 10% increase) | | 1.09 | - | 1.06 | 1.39 | 1.08 | - | 1.09 | 1.16 |
| PPDV (per 10% increase) | | 1.41 | - | 1.73 | - | - | - | 1.01 | - |
| Rural (Ref = Urban) | | 0.81 | - | 0.88 | - | - | - | - | - |
| Distance to clinic (per km) | | 1.09 | - | 1.09 | - | - | - | - | - |
| Distance to level 1 road (per km) | | 0.99 | - | 0.99 | - | - | - | - | - |
| Distance to level 2 road (per km) | | 0.95 | - | 0.97 | - | 0.92 | - | 0.94 | - |
| Distance to primary school (per km) | | - | - | - | - | 0.95 | - | 0.97 | - |
| Distance to secondary school (per km) | | - | - | - | - | - | - | - | - |

**Table S3: Area under the receiver operating characteristic curve (AUROC) estimated in the development and validation datasets.** AUROC values are averaged over 10 imputed datasets. AUROC in the development dataset was estimated through 10-fold cross-validation.

| | Men | | Women | |
|---|---|---|---|---|
| Model | **Development cv-AUROC** | **Validation AUROC** | **Development cv-AUROC** | **Validation AUROC** |
| Full | 0.74 | 0.72 | 0.71 | 0.68 |
| Individual covariates only | 0.73 | 0.73 | 0.71 | 0.68 |
| Age + geospatial covariates | 0.71 | 0.71 | 0.68 | 0.65 |
| Age + HIV prevalence | 0.71 | 0.68 | 0.68 | 0.64 |

**Figure S1: Percentage of high incidence area (≥ 3/100 PY) contained within varying percentages of the map with the highest predicted risk.** Incidence and predicted risk estimated for men and women combined from 2012-2019 and smoothed using a 2-dimensional Gaussian kernel. Full = no covariate restriction; Ind = only individual-level covariates; Age + Geo = only age group and geospatial covariates; Age + HIV prev = only age group and local HIV prevalence

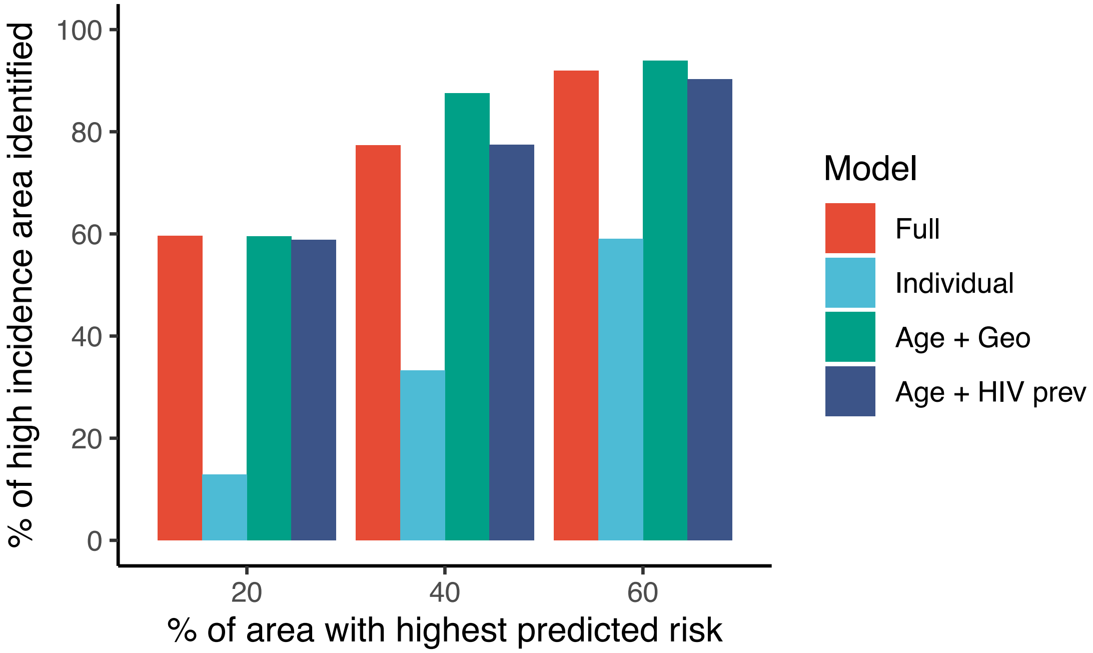

**Figure S2: Percentage of high incidence area (≥ 3/100 PY) contained within varying percentages of the map with the highest predicted risk, by incidence kernel bandwidth.** Incidence and predicted risk estimated for men and women combined from 2012-2019 and smoothed using a 2-dimensional Gaussian kernel. Full = no covariate restriction; Ind = only individual-level covariates; Age + Geo = only age group and geospatial covariates; Age + HIV prev = only age group and local HIV prevalence

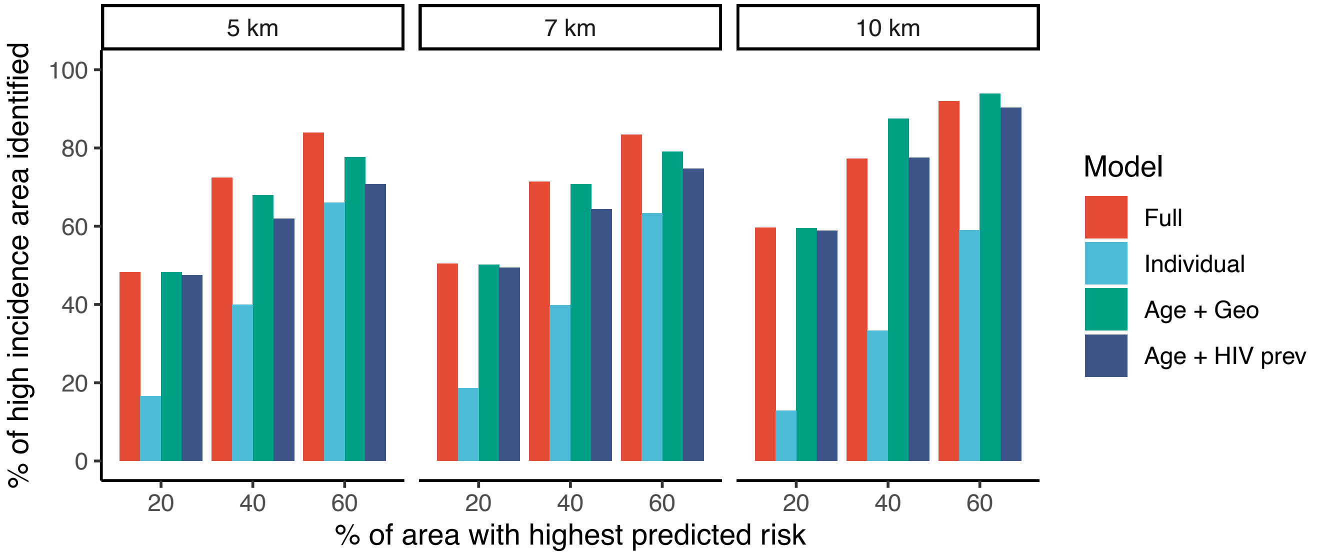

Supplement: ciac069_suppl_Supplementary_Material [file ciac069_suppl_supplementary_material.pdf]
